# Type 3 deiodinase activation mediated by the Shh/Gli1 axis promotes sepsis-induced metabolic dysregulation in skeletal muscles

**DOI:** 10.1093/burnst/tkae066

**Published:** 2025-01-28

**Authors:** Gang Wang, Tao Gao, Yijiang Liu, Jianfeng Duan, Huimin Lu, Anqi Jiang, Yun Xu, Xiaolan Lu, Xiaoyao Li, Yong Wang, Wenkui Yu

**Affiliations:** Department of Critical Care Medicine, Nanjing Drum Tower Hospital, Affiliated Hospital of Medical School, Nanjing University, No. 321 Zhongshan Road, Gulou District, Nanjing, Jiangsu 210008, China; The State Key Laboratory of Pharmaceutical Biotechnology, No. 22 Hankou Road, Gulou district, Nanjing, Jiangsu 210093, China; Department of Critical Care Medicine, Nanjing Drum Tower Hospital, Affiliated Hospital of Medical School, Nanjing University, No. 321 Zhongshan Road, Gulou District, Nanjing, Jiangsu 210008, China; The State Key Laboratory of Pharmaceutical Biotechnology, No. 22 Hankou Road, Gulou district, Nanjing, Jiangsu 210093, China; The State Key Laboratory of Pharmaceutical Biotechnology, No. 22 Hankou Road, Gulou district, Nanjing, Jiangsu 210093, China; Department of Critical Care Medicine, The Drum Tower Clinical Collage of Nanjing Medical University, No. 321 Zhongshan Road, Gulou District, Nanjing, Jiangsu 210008, China; Department of Critical Care Medicine, Nanjing Drum Tower Hospital, Affiliated Hospital of Medical School, Nanjing University, No. 321 Zhongshan Road, Gulou District, Nanjing, Jiangsu 210008, China; The State Key Laboratory of Pharmaceutical Biotechnology, No. 22 Hankou Road, Gulou district, Nanjing, Jiangsu 210093, China; Department of Critical Care Medicine, Nanjing Drum Tower Hospital, Affiliated Hospital of Medical School, Nanjing University, No. 321 Zhongshan Road, Gulou District, Nanjing, Jiangsu 210008, China; The State Key Laboratory of Pharmaceutical Biotechnology, No. 22 Hankou Road, Gulou district, Nanjing, Jiangsu 210093, China; Department of Critical Care Medicine, Nanjing Drum Tower Hospital, Affiliated Hospital of Medical School, Nanjing University, No. 321 Zhongshan Road, Gulou District, Nanjing, Jiangsu 210008, China; The State Key Laboratory of Pharmaceutical Biotechnology, No. 22 Hankou Road, Gulou district, Nanjing, Jiangsu 210093, China; Department of Critical Care Medicine, Nanjing Drum Tower Hospital, Affiliated Hospital of Medical School, Nanjing University, No. 321 Zhongshan Road, Gulou District, Nanjing, Jiangsu 210008, China; The State Key Laboratory of Pharmaceutical Biotechnology, No. 22 Hankou Road, Gulou district, Nanjing, Jiangsu 210093, China; Department of Critical Care Medicine, Nanjing Drum Tower Hospital, Affiliated Hospital of Medical School, Nanjing University, No. 321 Zhongshan Road, Gulou District, Nanjing, Jiangsu 210008, China; The State Key Laboratory of Pharmaceutical Biotechnology, No. 22 Hankou Road, Gulou district, Nanjing, Jiangsu 210093, China; Department of Critical Care Medicine, Nanjing Drum Tower Hospital, Affiliated Hospital of Medical School, Nanjing University, No. 321 Zhongshan Road, Gulou District, Nanjing, Jiangsu 210008, China; The State Key Laboratory of Pharmaceutical Biotechnology, No. 22 Hankou Road, Gulou district, Nanjing, Jiangsu 210093, China; The State Key Laboratory of Analytical Chemistry for Life Science and Jiangsu Key Laboratory of Molecular Medicine, Medical School of Nanjing University, No. 22 Hankou Road, Gulou district, Nanjing, Jiangsu 210093, China; Department of Critical Care Medicine, Nanjing Drum Tower Hospital, Affiliated Hospital of Medical School, Nanjing University, No. 321 Zhongshan Road, Gulou District, Nanjing, Jiangsu 210008, China; The State Key Laboratory of Pharmaceutical Biotechnology, No. 22 Hankou Road, Gulou district, Nanjing, Jiangsu 210093, China; Department of Critical Care Medicine, The Drum Tower Clinical Collage of Nanjing Medical University, No. 321 Zhongshan Road, Gulou District, Nanjing, Jiangsu 210008, China

**Keywords:** Type 3 deiodinase, Non-thyroidal illness syndrome, Sepsis, Sonic hedgehog, Muscle wasting

## Abstract

**Background:**

Non-thyroidal illness syndrome is commonly observed in critically ill patients, characterized by the inactivation of systemic thyroid hormones (TH), which aggravates metabolic dysfunction. Recent evidence indicates that enhanced TH inactivation is mediated by the reactivation of type 3 deiodinase (Dio3) at the tissue level, culminating in a perturbed local metabolic equilibrium. This study assessed whether targeted inhibition of Dio3 can maintain tissue metabolic homeostasis under septic conditions and explored the mechanism behind Dio3 reactivation.

**Methods:**

A retrospective clinical study was conducted to investigate the attributes of rT3. The expression of Dio3 was detected by immunoblotting, immunofluorescence, and immunohistochemical staining in tissues extracted from CLP-induced septic rats and human biopsy samples. In addition, the effect of Dio3 inhibition on skeletal muscle metabolism was observed in rats with targeted Dio3 knockdown using an adeno-associated virus. The effectiveness of Sonic hedgehog (Shh) signaling inhibition on systemic TH levels was observed in CLP-induced septic rats receiving cyclopamine. The mechanisms underlying such inhibition were explored using immunoblotting, RNA-seq, and chromatin immunoprecipitation–qPCR assays.

**Results:**

The main product of Dio3, rT3, is strongly associated with organ function. Early sepsis leads to significant upregulation of Dio3 in the skeletal muscles and lung tissues of septic rats. The targeted inhibition of Dio3 in skeletal muscle restores TH responsiveness, prevents fast-to-slow fiber conversion, preserves glucose transporter type 4 functionality, and maintains metabolic balance between protein synthesis and proteolysis, which leads to preserved muscle mass. The reactivation of Dio3 is transcriptionally regulated by the Shh pathway induced by the signal transducer and activator of transcription 3.

**Conclusions:**

The suppression of Dio3 restores tissue TH actions, attenuates proteolysis, and ameliorates anabolic resistance in the skeletal muscles of septic rats, thereby improving local metabolic homeostasis. Our results provide insights into the mechanisms of Dio3 reactivation and its critical role in local metabolic alterations induced by sepsis, while also suggesting novel targets aimed at ameliorating tissue-specific metabolic disorders.

## Graphical Abstract

**Graphical Abstract:**
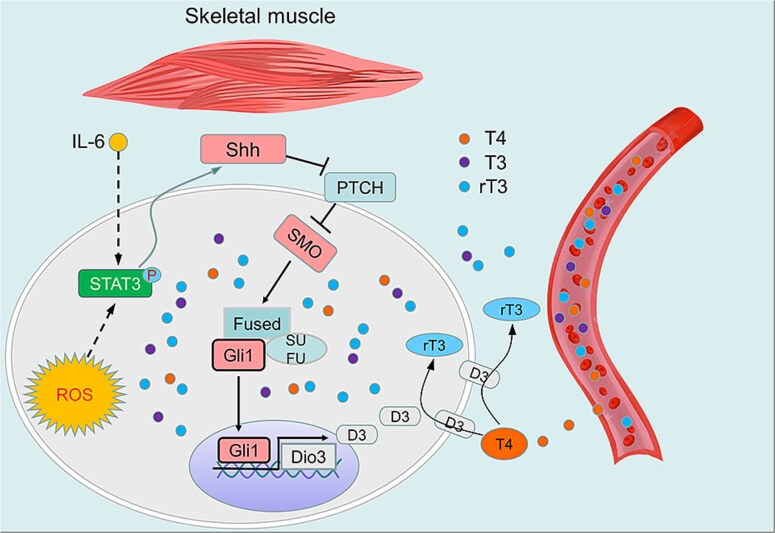

Highlights

- Dio3 is robustly induced in skeletal muscle and lung tissues upon early sepsis.
- The Shh/Gli1 axis facilitates the reactivation of Dio3 in skeletal muscle.
- Targeted inhibition of Dio3 preserves metabolic homeostasis in the muscle of septic rats.
- Suppression of Dio3 ameliorates the systemic alterations of thyroid hormone.

## Background

Thyroid hormones (TH) support the body’s normal growth and organ maturation, and play pivotal roles in the modulation of basal metabolic rate and mitochondrial quality control [1]. In the milieu of critical illness, marked perturbations in the TH axis are evident, which are characterized by reduced systemic levels of triiodothyronine (T3) and free T3 (fT3), concomitant with an elevation of reverse T3 (rT3). The adjustments of the TH axis to various pathological conditions such as burns, sepsis, and trauma are known as ‘non-thyroidal illness syndrome (NTIS)’ [2]. This syndrome is prevalent among critically ill patients, and the T3 levels are inversely associated with poor prognosis [3].

NTIS is considered an adaptation in response to acute stress. That is, the body attempts to reduce T3 levels, thereby decreasing the basal metabolic rate and energy expenditure, to promote survival [4]. However, specialists in the critical care department hold a different view. The energy expenditures of critically ill patients are notably increased, especially in patients with burns [5]. A more reasonable explanation is that the body suppresses physiological metabolism to support the production of inflammatory cytokines, the synthesis of acute-phase proteins, and the energy supply to vital organs. However, this protective method results in persistent anabolic resistance and enhanced proteolysis, which may lead to various organ dysfunctions, including diaphragm weakness, respiratory muscle fatigue, and skeletal muscle atrophy [6]. In addition, TH plays pivotal roles in clotting, endothelial function, immune system, and mitochondrial quality control [7]. Thus, numerous physicians have explored the rectification of low T3 state to ameliorate metabolic disarrangements and organ dysfunction in critically ill patients by replacement therapy. Although preclinical investigations achieved satisfying results, such therapeutic strategies have failed to demonstrate a decrease in mortality in critically ill patients [2].

At present, the mechanism underlying the occurrence of this therapeutic ineffectiveness remains unknown. Notably, illness elevates hypothalamic T3 levels via upregulated local type-2 deiodinase (Dio2) activity, thereby suppressing thyrotropin-releasing hormone (TRH) neuronal function and attenuating central TH axis modulation [8]. Therefore, T3 replacement therapy may further suppress TRH neurons, leading to more severe metabolic disarrangements caused by the activated sympathetic nervous system [9]. Moreover, deiodinases, transporters, and receptors modulate tissue TH actions, which is an important consideration as diminished tissue sensitivity to TH is well documented [10] and may hinder the effectiveness of replacement therapy. Therefore, in addressing the low T3 state and restore TH actions, the regulatory mechanisms determining TH concentrations at the tissue level must be considered.

Notably, tissue T3 levels are intricately orchestrated by the action of deiodinases. Dio2 contributes to this meticulous control by facilitating outer ring deiodination, thereby converting thyroxine (T4) into the more biologically active T3. In contrast, type-3 deiodinase (Dio3) catalyzes inner ring deiodination of T4, leading to its inactivation by converting T4 into biologically inactive rT3 [11]. Type-1 deiodinase (Dio1) is primarily expressed in the liver and kidney, and it is capable of inner and outer ring deiodination. Consistent evidence from past investigations has demonstrated that Dio1 and Dio2 activities are promptly inhibited in various tissues during critical illness, whereas the activity of Dio3 is robustly increased [12]. The induced expression of Dio3 robustly inactivates TH signaling in skeletal satellite cells [13]. Inhibiting the expression of Dio3 in neuronal cells and cardiomyocytes accelerates energy expenditure [14]. These findings underscore the crucial contribution of Dio3 to the TH actions and local metabolic homeostasis of tissues. However, the profile of Dio3 expression as well as the mechanisms underlying the induction of Dio3 remain largely unknown. Moreover, the effectiveness of Dio3 inhibition on preserving local metabolic homeostasis under septic conditions remains to be investigated.

In addressing these challenges, we used a cecal ligation and puncture (CLP) induced septic rat model and found that Dio3 is mainly induced in skeletal muscles and lung tissues 24 h after CLP modelling. Targeted Dio3 inhibition in the tibialis anterior (TA) muscle prevents fast-to-slow myofiber conversion and maintains glucose transporter type 4 (GLUT4) membrane translocation, which indicates preserved local glucose metabolism. In addition, Dio3 inhibition restored anabolic hallmarks and repressed catabolic ones, leading to preserved muscle mass. By performing RNA-seq analysis and immunoblotting, the robust activation of Sonic hedgehog (Shh) signaling was confirmed, which facilitates the induction of Dio3 by enhancing the binding of Glioma Associated Oncogene Family Zinc Finger 1 (Gli1) to the promoter of Dio3. Finally, the activation of Shh is the signal transducer and activator of transcription 3 (STAT3), which is dependent on the IL-6 and ROS involved in this process. Our results clarify the critical role of Dio3 in local metabolic alterations and propose novel interventions targeting STAT3/Shh/Gli1 signaling aimed at ameliorating tissue-specific metabolic disorders under septic conditions.

## Methods

### Human study design and samples

This observational study recruited adult patients (age ≥ 18 years) presenting with critical illness and admitted to the Intensive Care Unit (ICU) at Nanjing Drum Tower Hospital. Eligibility for inclusion requires individuals to receive at least one type of life-supportive therapy for organ dysfunction. Those with a documented history of thyroid disease, detected potential thyroid disease, or established hypothalamic injury were not eligible. To determine the initial severity of illness, we used the Acute Physiological and Chronic Health Evaluation II (APACHE II) score, and organ dysfunction was measured using the Sequential Organ Failure Assessment (SOFA) scoring system. Blood samples were taken within 8 h of ICU admission for conventional hematological assessment and measurement of TH concentrations. Other relevant biological data was also collected from electronic health records for later analysis. Ten critically ill patients (range 33–71 years; n = 5 male, n = 5 female) underwent skeletal muscle biopsy by senior specialists under ultrasound. The samples were collected for further immunoblotting, immunohistochemistry (IHC), or immunofluorescence (IF) assays. All clinical data and skeletal muscle samples were collected with written informed consent from patients and following the guidelines approved by the ethics boards of the Nanjing Drum Tower Hospital (Ethics number: 2023–427-03).

### Animal experiments design

Wild-type Sprague Dawley 8-week-old male rats (n = 42), and 4-week-old male rats (n = 20) were purchased from Charles River Company (Nanjing, China). The rats were maintained in a specific pathogen-free environment with regular lighting conditions (12 h light/dark cycles) under 24°C. Free access to water and food was provided. CLP was selected as the establishment of sepsis modeling and was carried out as previously described. Targeted inhibition of Dio3 was conducted by local injection with pAAV-EGFP-U6-shDio3 (BOIO-HYKY-220726011-DAAV,1^*^10^11^ genomic copies/rat) to silence Dio3 in the TA muscle. pAAV-EGFP-U6-shDio3 (AAV-shDio3) were constructed by OBiO Technology (Shanghai, China), shDio3: GCGCGACGTTGACTTCCTTAT. The experiment was conducted in three parts: in the first part, rats were divided into a sham group (n = 6) and CLP group (n = 10); In the second part, rats were divided into three groups: a shNC + sham group (n = 6), shNC + CLP group (n = 8), and shDio3 (shD3) + CLP group (n = 8). Rats were either injected with AAV-shDio3 or AAV-vector for targeted silencing of Dio3 in TA; In the third part, rats were randomly divided into a sham group (n = 6), CLP group (n = 10), and CLP + Cyclopamine (HY- 17024, MCE, 40 mg/kg, intraperitoneal injection 2 h before CLP modeling) group (n = 10). 24 h after CLP modeling (48 h for rats in part 2), rats were sacrificed, and blood and tissue samples were harvested by surgical procedure. Part of the tissue samples were fixed in 4% paraformaldehyde, and the remaining were stored at −80°C for molecular analysis. All animal experiments have followed the guidelines of ARRIVE and approved by the Ethics Committee of Nanjing Drum Tower Hospital (Ethics number: 2022AE02004).

### Cell culture and treatments

The murine myoblast cell line C2C12 (obtained from Pricella Biotechnology, Wuhan, China), human embryonic kidney cells HEK-293 T (generously gifted by professor Wang Yong, Nanjing University Medical School), human hepatocellular carcinoma cells HepG2 (obtained from Pricella Biotechnology, Wuhan, China), and human lung adenocarcinoma cells PC9 (obtained from Pricella Biotechnology, Wuhan, China) were propagated in Dulbecco’s Modified Eagle Medium (DMEM, Gibco, USA) supplemented with 10% fetal bovine serum (Hyclone, USA) and 1% penicillin–streptomycin solution (Gibco, USA). Cultivation occurred under a humidified atmosphere containing 5% CO2 balanced with air at a stable temperature of 37°C. The differentiation of C2C12 cells proceeded according to previously established protocols. To establish an in vitro model of inflammation, lipopolysaccharide (LPS) was introduced to the culture medium. In alignment with the experimental design, various agents, including rShh protein (P7325, Beyotime, China), Gant61 (HY-13901, MCE, USA), recombinant interleukin-6 (IL-6) (P5925, Beyotime, China), Stattic (S7024, Selleck, USA), and N-acetylcysteine (NAC) (STV2524, Beyotime, China) were employed.

### Immunohistochemistry

Human and murine skeletal muscle and lung samples were sectioned at 4 μm, and stained for Shh (A12695, Abclonal, China) or Dio3 (A6900, Abclonal, China). These sections were then incubated with a goat antirabbit IgG-HRP secondary antibody. A Leica optical microscope captured the resultant images (Leica Microsystems, Germany).

### Immunofluorescence

Lung and skeletal tissues from rats were sectioned and stained for Shh (A12695, Abclonal, China), MYH1(GB112130, Servicebio, China), MYH7 (GB112131, Servicebio, China), and GLUT4 (A7637, Abclonal, China) by standard protocols. Nuclei were highlighted with DAPI counterstaining and imaged using an Olympus inverted fluorescence microscope (IXplore standard, Japan).

### Immunoblotting

Immunoblotting assays of tissues or cell lysates were performed following established protocol. 1: 1000 diluted fresh primary antibodies or anti–β-Tubulin (1: 2000) in TBST, and 1: 20000 diluted fresh goat antirabbit IgG-HRP (A21020, Abbkine, China), and goat antimouse IgG-HRP (A21010, Abbkine, China) in TBST. The blots were imaged using a chemiluminescence imaging system (Tanon, China). Densitometric analysis was performed with ImageJ. All primary antibodies used were listed in Table S1 (see online supplementary material).

### Real-time quantitative PCR

Total RNA from murine tissues and cells was isolated using the RNAeasy Animal Isolation Kit (R0026, Beyotime, China). cDNA synthesis followed using Vazyme’s reverse transcription kit (R323–01, Vazyme, China). Subsequent RT-PCR was conducted on an ABI Viia 7 system using ChamQ SYBR Color qPCR Master Mix (Q431–02, Vazyme, China) with primers listed in Table S2. Ct values were ascertained for quantification, employing the 2^−ΔΔCT method, with β-actin serving as the internal reference.

### Chromatin immunoprecipitation

Chromatin immunoprecipitation (ChIP) assay was conducted using a commercial enzymatic ChIP kit (P2083S, Beyotime, China). C2C12 myotube nuclear lysates, post 1% formaldehyde cross-linking, were sonicated to fragment chromatin, followed by immunoprecipitation with Gli1 (sc-515751, Santa Cruz, USA) antibody or control IgG. Amplification of target genomic regions from both input and immunoprecipitated samples was performed via both PCR and real-time quantitative PCR. PCR products underwent electrophoresis on 2% agarose gels and were visualized with a BG-gdsAUTO550 gel imaging system (Baygene, China), then quantified using ImageJ. Primers used for the Dio3 promoter putative Gli1 binding motif were 5’-GTCTCCCGCCAATTCAAG-3′ (sense) and 5’-CTTCACCTTCTCCGACCA-3′ (antisense).

### Thyroid hormone detection

Serum total T4, T3, and rT3 concentrations were quantified with a commercially available radioimmunoassay kit (Beijing North Institute of Biotechnology, China) and performed by experts from the Clinical Laboratory Department of Drum Tower Hospital.

### Intraperitoneal glucose tolerance test

Intraperitoneal glucose tolerance test was performed in the morning following CLP modeling by administering a bolus of 20% glucose (2 g/kg, i.p.). Blood samples were serially collected from the tail vein at 0, 15, 30, 60, and 120 min postinjection for glucose level measurement.

### RNA-seq analysis

C2C12 cells of control or 4 h LPS exposure were collected for RNA isolation. They proceeded to library construction and RNA-Seq at Kaitaibio (Hangzhou, China) using paired-end 150 bp reads on an Illumina Novaseq 6000. FASTQ files from sequencing were quality controlled and adapter trimmed using FASTQC and mapped to the mm39 reference genome using the Hisat2. Putative PCR duplicates were marked and removed with SAM tools for downstream analysis. Gene expression levels were determined by the number of fragments per kilobase per million reads, similar to a previously described procedure. Briefly, reads overlapping exonic regions of Ensembl mm39 known genes were determined using the Merge function of the Stringtie. Differentially expressed genes were determined using edgeR with a log2FC > 1 and padj < 0.05 determining significance.

### Statistical analysis

Thorough statistical analyses were conducted using the advanced GraphPad Prism 8 software and SPSS statistics 26. To ensure accuracy, the normality of data and homogeneity of variance were assessed using rigorous statistical tests such as Shapiro–Wilk and Levene’s, respectively. For multiple comparison analyses, we utilized either one- or two-way ANOVA, followed by Tukey’s post-hoc test for specific pairwise comparisons. Binary variables were compared using the t-test. For correlation analyses, Spearman correlation analysis was used. The data is presented either as mean ± SD or through visually interpretive box-and-whisker plots. A significance threshold of *p* < 0.05 was implemented to determine statistical significance.

## Results

### rT3 is a potent indicator of organ function

**Figure 1:**
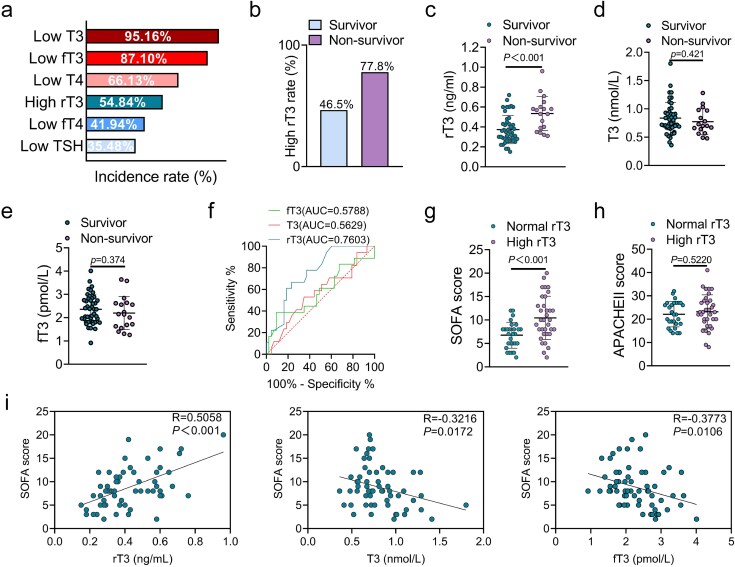
rT3 as a potent indicator of organ function. (**a**) the incidence rate of low T3, T4, fT3, fT4, and TSH levels, as well as high rT3 level (the reference values for defining high or low TH-axis parameters are provided by the clinical laboratory at Nanjing drum tower; n = 62). (**b**) Incidence rate of high rT3 level in survivors (n = 43) and non-survivors (n = 19). (**c**) rT3 levels in the same setting as in (b). (**d**) T3 levels in the same setting as in (b). (**e**) fT3 levels in the same setting as in (b). (**f**) Mortality risk ROC curve and AUC score with T3, fT3, and rT3 parameters (n = 62). (**g**) SOFA scores of patients in the normal rT3 group (n = 28) and the high rT3 group (n = 34). (**h**) APACHE II scores of patients in the normal rT3 group (n = 28) and the high rT3 group (n = 34). (**i**) Correlation analysis among rT3, T3, fT3, and SOFA scores (n = 62). ^*^P < 0.05. Mann–Whitney test for (c, d), two-tailed Student’s unpaired t-test for (e, g, and h), ROC curve analysis for (f), or Spearman’s rank correlation analysis for (i). Quantitative data are represented as mean ± SD. *NS* not significant, *T3* triiodothyronine, *T4* thyroxine, *SOFA* sequential organ failure assessment, *APPACHE II* acute physiology and chronic health evaluation

In the present study, we found elevated baseline rT3 levels in 54.84% of patients, which is lower than the incidence rate of low T3 (95.16%), fT3 (87.10%), and T4 (66.13%; Figure 1a). To comprehensively understand the characteristics of rT3, the patients were divided into survivor groups and non-survivor groups. The results indicated that the incidence of a high rT3 state is higher in deceased patients (Figure 1b). Compared with T3 and fT3, rT3 levels are higher in deceased patients, indicating that the baseline rT3 level may be intimately associated with poor prognosis (Figure 1c–e; Table 1). The results of receiver operating characteristic (ROC) curve analysis corroborated that rT3 (AUC = 0.7603) can better predict mortality than T3 (AUC = 0.5629) and fT3 (AUC = 0.5788; Figure 1f). To explain this result, we further divided the patients into a normal rT3 group and a high rT3 group. Results indicated that patients with higher rT3 levels tend to achieve higher SOFA scores (Figure 1g) instead of APACHE II scores (Figure 1h). Correlation analysis results showed a stronger correlation between rT3 levels and SOFA scores than T3 and fT3 (Figure 1i), indicating that rT3 level is closely correlated with organ function. Collectively, the main product of Dio3, rT3, serves as a potent biomarker of poor prognosis and a strong indicator of organ function.

**Table 1:** Baseline characteristics of patients within 24 h following admission to ICU

	**Total**	**Survivors**	**Non-survivors**	***P***
Number of patients	62	44	18	
**Demographics**				
Age, years, mean ± SD	62.1±15.8	58.5±16.2	70.1±1v0.9	0.004
Male, n (%)	45 (72.6)	32 (72.7)	13 (72.2)	0.968
**Principal diagnosis (%)**				
Neurologic disease, n (%)	4(6.45)	2(4.55)	2(11.11)	0.573
Digestive disease, n (%)	9(14.52)	5(11.36)	4(22.22)	0.427
Poisoning, n (%)	2(3.22)	2(4.55)	0	0.581
Infectious disease and/or sepsis, n (%)	26(41.94)	17(36.36)	9(50.00)	0.257
Trauma, n (%)	18 (29.03)	16 (36.36)	2 (11.11)	0.065
Other, n (%)	3 (4.84)	2 (4.55)	1 (5.56)	0.866
APACHE II score, median (IQ1-IQ3)	22.5 (18.3–27.0)	20.0 (16.8–26.3)	26.0 (23.3–28.8)	0.0103^a^
SOFA score, median (IQ1-IQ3)	8 (5.3–11.8)	8 (5.0–10.0)	9.5 (8.0–12.0)	0.0574
IL-6, pg/ml, median (IQ1-IQ3)	58.6 (28.0–153.0)	48.8 (24.7–122.0)	100.3 (53.0–180.5)	0.9448
PCT, ng/ml, median (IQ1-IQ3)	0.97 (0.21–2.63)	0.83 (0.19–2.56)	1.27 (0.52–7.03)	0.3550
**Thyroid hormone parameters**				
TSH, mIU/L, median (IQ1-IQ3)	0.46 (0.23–1.42)	0.61 (0.26–1.52)	0.32 (0.15–0.74)	0.4958
T3, nmol/L, median (IQ1-IQ3)	0.74 (0.66–0.94)	0.78 (0.67–0.93)	0.70 (0.64–0.97)	0.4207
T4, nmol/L, median (IQ1-IQ3)	56.0 (45.8–71.7)	55.3 (47.1–73.2)	60.7 (41.5–70.1)	0.7234
fT3, pmol/L, median (IQ1-IQ3)	2.21 (1.81–2.74)	2.37 (1.92–2.77)	2.15 (1.65–2.61)	0.3736
fT4, pmol/L, median (IQ1-IQ3)	12.7 (11.0–14.9)	12.5 (10.9–13.9)	13.5 (12.0–16.3)	0.0636
rT3, ng/ml, median (IQ1-IQ3)	0.38 (0.29–0.57)	0.35 (0.28–0.44)	0.55 (0.39–0.60)	0.0003^*^

a *P* < 0.05, Quantitative data are represented as mean ± SD. Comparisons were done using Fisher’s exact test for categorical variables and Mann–Whitney U test for continuous variables. *SOFA* sequential organ failure assessment, *APPACHE II* Acute Physiology and Chronic Health Evaluation, *IL-6* interleukin 6, *PCT* procalcitonin, *TSH* thyroid stimulating hormone, *T3* triiodothyronine, *T4* thyroxine, *rT3* reverse, T3, *ICU* Intensive Care Unit

### Dio3 expression increased mainly in skeletal muscles and lung tissues upon early sepsis

**Figure 2:**
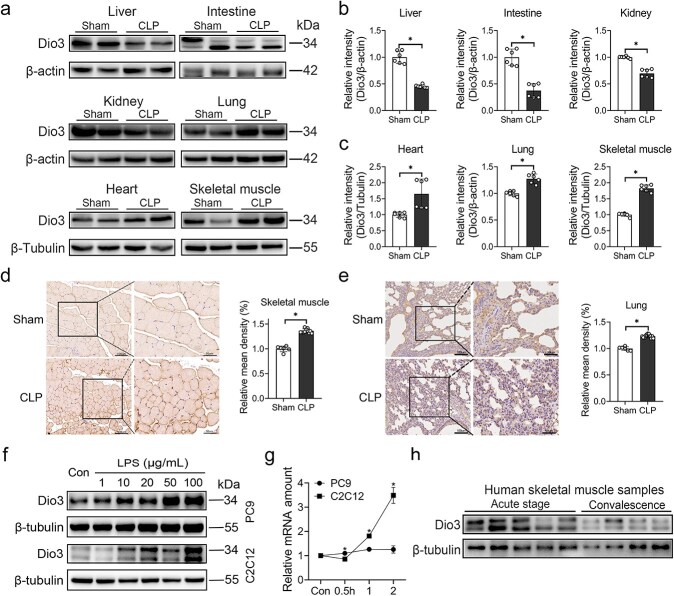
Dio3 expression increased mainly in skeletal muscles and lung tissues upon early sepsis. (**a**) Representative immunoblots of Dio3 expression in primary peripheral tissues, including the liver, intestine, kidney, heart, lung, and skeletal muscles, of control rats or rats subjected to CLP modelling (n = 6). (**b, c**) Quantitative analysis of (a). (**d**) Representative images of Dio3 IHC staining in skeletal muscles of rats with or without CLP modelling and their quantification (n = 6; scale bar: 50 μm&100 μm). (**e**) Representative images of Dio3 IHC staining in lung tissues of rats with or without CLP modelling and their quantification (n = 6; scale bar: 50 μm&100 μm). (**f**) Immunoblotting demonstrating the expression of Dio3 in PC9 and C2C12 cells exposed to gradient LPS concentrations for 24 h (0, 1, 10, 20, 50, and 100 μg/ml; n = 3). (**g**) Quantitative RT-PCR analysis of Dio3 mRNA expression in PC9 and C2C12 cells treated with 100 μg/ml LPS (n = 3). (**h**) Immunoblotting demonstrating the expression of Dio3 in human skeletal muscle biopsy samples (acute stage, biopsy taken within 1 week of admission; convalescence, biopsy taken shortly before discharge during the convalescent stage). ^*^P < 0.05; NS, not significant. Two-tailed Student’s unpaired t-test and Mann–Whitney test for (b, c) or two-tailed Student’s unpaired t-test for (d, e and g). Quantitative data are represented as mean ± SD. *NS* not significant, *Dio3* type 3 deiodinase, *CLP* cecal ligation and puncture, *LPS* lipopolysaccharide, *IHC* immunohistochemistry

Given that elevated rT3 levels correlate positively with Dio3 activity [15], Dio3 expression was further evaluated in six major peripheral tissues from CLP-induced septic rats. Our findings indicated that Dio3 expression was inhibited in the liver, intestine, and kidney (Figure 2a and b) but significantly induced in the heart, lung, and skeletal muscle 24 h after CLP modeling (Figure 2a and c). Based on a previous study, although Dio3 was strongly induced in the heart tissues of mice that underwent myocardial infarction, little alteration in T3 levels was detected [16]. Thus, the systemic TH axis was more likely to be affected by the lung or skeletal muscle. IHC staining confirmed the evident increase of Dio3 expression level in skeletal muscles and lung tissues induced by CLP (Figure 2d and e). To further explore the characteristics of Dio3 expression in the lung and skeletal muscles, inflammatory cell models were established in the mouse C2C12 cell line and human lung adenocarcinoma PC9 cell line. The results demonstrated a dose-dependent increase in Dio3 expression in PC9 and C2C12 cell lines upon LPS treatment (Figures 2f and S1a). Considering that rT3 levels can rise remarkably as early as 6 h poststress [17], the increase in Dio3 expression likely precedes the changes in rT3 levels. Accordingly, Dio3 expression was detected in C2C12 and PC9 cells following LPS exposure in a time-course manner. The results indicate that the mRNA expression level of Dio3 increased significantly in PC9 and C2C12 cells, but the increment of Dio3 expression in C2C12 cells is more notable than that in PC9 cells after 1-h exposure to LPS (Figure 2g). In addition, a robustly induced expression of Dio3 was detected in human skeletal muscle biopsy samples at the acute stage of sepsis, which is consistent with previous research (Figures 2h and S1b) [18].

### Targeted inhibition of Dio3 ameliorates sepsis-induced metabolic disruption in skeletal muscles

**Figure 3:**
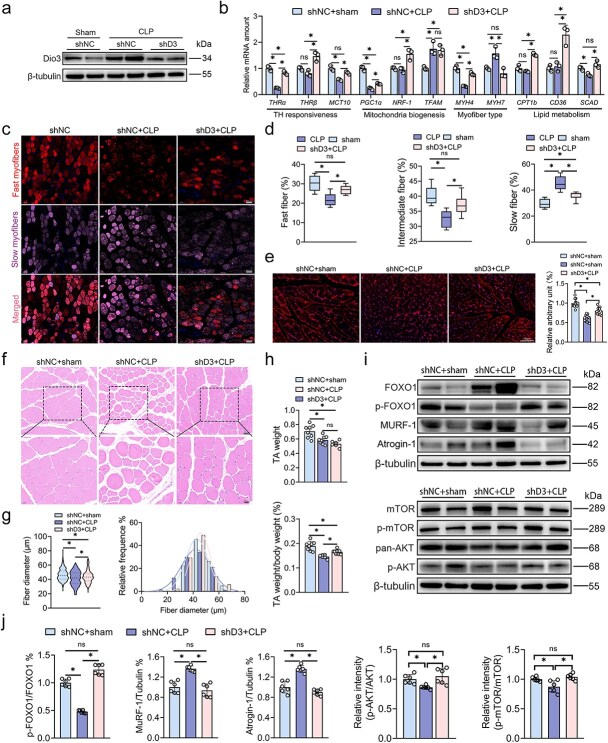
Targeted inhibition of Dio3 ameliorates sepsis-induced metabolic disruption in skeletal muscles. (**a**) Representative immunoblots of Dio3 in the TA of rats belonging to the shNC + sham, shNC + CLP, and shD3 + CLP groups (n = 6). (**b**) Quantitative RT-PCR analysis of T3-responsive genes in samples in the same setting as in (a; n = 3). (**c**) Representative images of IF staining of MYH7 and MYH4 in TA (scale bar: 50 μm) of rats in the same setting as in (a; n = 6). (**d**) Quantitative analysis of fiber proportion in (c) by ImageJ. (**e**) Representative images of the IF staining of Glut4 in TA (scale bar: 100 μm) of rats in the same setting as in (a) and their quantification (n = 6). (**f**) Representative images of the HE staining of skeletal muscles in the same setting as in (a; n = 6; scale bar: 20 μm&50 μm). (**g**) Quantitative analysis of the fiber diameters in (f) by ImageJ. (**h**) Weight of TA and its proportion to body weight (n = 6). (**i**) Representative immunoblots of the expression of catabolic hallmarks (FOXO1, p-FOXO1, MuRF1, and ATROGIN1) and anabolic hallmarks (mTOR, p-mTOR, AKT, and p-AKT) in the skeletal muscle tissues of rats in the same setting as in (a; n = 6). (**j**) Quantification analysis of immunoblots in (i; n = 6). ^*^P < 0.05. Two-tailed Student’s unpaired t-test and Mann–Whitney test for (g, j) or one-way ANOVA followed by Tukey’s multiple comparisons tests for (b, d, e, h, and j). Quantitative data are represented as mean ± SD. *NS* not significant, *Dio3* type 3 deiodinase, *CLP* cecal ligation and puncture, *LPS* lipopolysaccharide, *THR* thyroid hormone receptor, *MCT10* solute carrier family 16 member 10, *PGC1α* peroxisome proliferator-activated receptor gamma coactivator 1α, *NRF1* nuclear respiratory factor 1, *TFAM* transcription factor a mitochondrial, *MYH* myosin heavy chain, *CPT1b* carnitine Palmitoyltransferase 1B, *SCAD* short chain acyl-CoA dehydrogenase, *TA* tibialis anterior, *FOXO1* Forkhead box O1, *MURF-1* muscle-specific RING finger protein 1, *Atrogin1* muscle atrophy F-box protein, *mTOR* mechanistic target of rapamycin kinase, *AKT* protein kinase B

Previous investigations have identified that the disrupted metabolic balance is a major hallmark of skeletal muscles when facing sepsis, which leads to continuous muscle mass loss and weakness. In exploring the impact of Dio3 induction in skeletal muscles under septic conditions, Dio3 expression was inhibited in rats’ TA by utilizing AAV (Figure 3a). The inhibition of Dio3 restored the expression of TH-responsive genes, including THRα, THRβ, MCT10, PGC1α, MYH4, MYH7, and CPT1b, indicating restored TH actions (Figure 3b). The qPCR and IF results collectively indicate that CLP modelling promotes the conversion of fast-to-slow myofibers, whereas the inhibition of Dio3 reverses this process (Figure 3c and d). Given the glycolytic pattern of fast twitch myofibers, we also examined the expression of GLUT4, which is the main glucose transporter in skeletal muscles. The results of IF staining of GLUT4 reveal that Dio3 inhibition restored GLUT4 expression and maintained its localization at the cell membrane (Figure 3e). The HE staining results demonstrated a significant increase in skeletal muscle fiber diameters following Dio3 inhibition compared with the CLP group (Figure 3f and g). In addition, although the absolute weight of the TA did not significantly differ between the two groups, the ratio of TA weight to body weight remarkably increased post-Dio3 inhibition compared with the CLP group (Figure 3h). Finally, we evaluated the major protein markers of protein synthesis and proteolysis. The results indicate that Dio3 inhibition restored the expression levels of anabolic markers, including p-mTOR/mTOR and p-AKT/AKT, and suppressed the expression of catabolic proteins, including FOXO1, MuRF-1, and Atrogin-1 (Figure 3i and j). Collectively, the inhibition of Dio3 maintains the glycolytic phenotype, preserves muscle mass, and ameliorates the metabolic disruption induced by sepsis in skeletal muscles.

### Shh signaling is involved in the reactivation of Dio3

**Figure 4:**
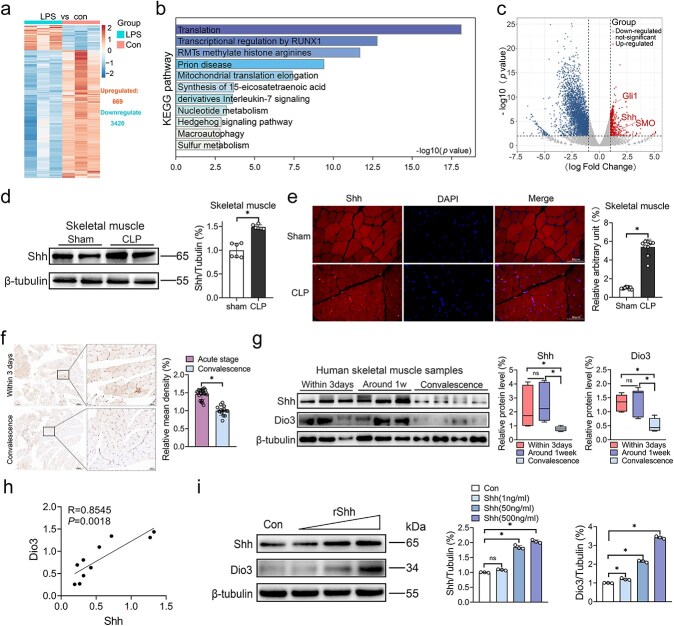
Shh signaling is involved in the reactivation of Dio3. (**a**) Comparative analysis of altered genes between the con group and C2C12 cells exposed to LPS for 4 h (one technical replicate of three biological replicates per group). (**b**) Analysis of the KEGG pathway from upregulated genes in the same setting as in (a). (**c**) Analysis of volcano plot from altered genes in the same setting as in (a). (**d**) Representative immunoblots of Shh expression in the skeletal muscle tissues of control rats or rats subjected to CLP modelling and their quantification (n = 6). (**e**) Representative images of the IF staining of Shh in skeletal muscles (scale bar: 50 μm) and quantification of Shh-positive area per selective field in rats with or without CLP modelling (n = 6). (**f**) Immunohistochemical staining of Dio3 in skeletal muscle biopsy samples from patients (scale bar: 50 μm&100 μm) and its quantification (n = 10; acute stage, biopsy taken within 1 week of admission; convalescence, biopsy taken shortly before discharge during the convalescent stage). (**g**) Immunoblotting demonstrating the expression of Shh and Dio3 in biopsy samples from the skeletal muscle of critically ill patients and its quantification (n = 10). (**h**) Spearman’s rank correlation analysis for the expression of Shh and Dio3 in the skeletal muscles of critically ill patients (n = 10). (**i**) Immunoblotting demonstrating the expression of Shh and Dio3 in C2C12 cells treated with gradient concentrations of rShh (0, 10, 200, and 500 ng/ml; n = 3) and its quantification. ^*^P < 0.05. Two-tailed Student’s unpaired t-test for (d), Mann–Whitney test for (e, f, and g), spearman correlation analysis for (h), or one-way ANOVA followed by Tukey’s multiple comparisons test for (i). Quantitative data are represented as mean ± SD. *NS* not significant, *LPS* lipopolysaccharide, *Shh* sonic hedgehog, *CLP* cecal ligation and puncture, *Dio3* type 3 deiodinase

In elucidating the mechanisms involved in Dio3 reactivation in the early stages of sepsis, we conducted RNA-seq analysis in C2C12 myoblasts following 4 h of LPS exposure. Although the LPS exposure time was shortened, marked changes in gene expression were still detected. The results showed 669 upregulated genes and 3420 downregulated ones compared with the control group (Figure 4a). The upregulated genes were subsequently subjected to KEGG pathway enrichment analysis. After 4 h of LPS stimulation, the results of KEGG enrichment analysis indicate that the upregulated genes are predominantly enriched in processes related to translation and posttranslational modification, inflammatory response, nucleoside metabolism, and organelle processes. Interestingly, the hedgehog signaling cascade was also been enriched, which was reported to be involved in the regulation of Dio3 (Figure 4b). Volcano plot analysis revealed a notable upregulation of genes involved in the Shh pathway, including Shh, smoothened (SMO), and Gli1 (Figure 4c). Given the pivotal role of the Shh pathway in governing Dio3 regulation in basal cell carcinomas (BCC) [19], we hypothesized that the activation of the Shh pathway may also remarkably contribute to the reactivation of Dio3 within an inflammatory milieu. To confirm this hypothesis, we examined the expression level of Shh in skeletal muscles and lung samples from septic rats. The data derived from immunoblotting and IF assays demonstrated a significant increment of Shh expression level in septic tissues (Figures 4d, e, S2a, and S2b). Immunohistochemical staining of Shh in skeletal muscle biopsy samples from patients with sepsis further confirmed its upregulation compared with convalescence ones (Figure 4f). The immunoblotting results also demonstrate a marked increase of Shh and Dio3 expression level in samples of patients in the acute phase (within 1 week of admission), with a substantial reduction during the convalescent stage (shortly before discharge during the convalescent stage; Figure 4g). In addition, a significant correlation between Shh and Dio3 expression was observed, with an R = 0.8545 (Figure 4h). Moreover, treating C2C12 cells with gradient concentrations of recombinant Shh (rShh) induced Dio3 expression in a dose-dependent manner (Figure 4i). Collectively, these results indicate that the activation of the Shh pathway underlies the reactivation of Dio3 under septic conditions.

### Shh regulates Dio3 expression via Gli1 in myoblasts under an inflammatory backdrop

**Figure 5:**
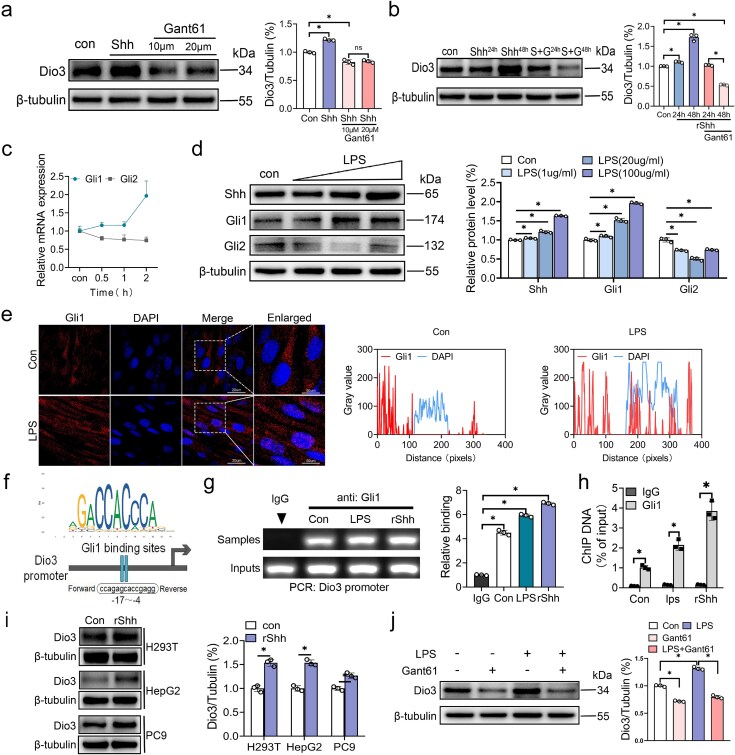
Shh regulates Dio3 expression via Gli1 in myoblasts under an inflammatory backdrop. (**a**) Immunoblotting demonstrating the expression of Dio3 in C2C12 cells treated with rShh (500 ng/ml) or Gant61 (10 or 20 μM) and its quantification (n = 3). (**b**) Immunoblotting demonstrating the expression of Dio3 in C2C12 cells treated with rShh (500 ng/ml) or 10 μm Gant61 for 24 or 48 h (n = 3) and its quantification. (**c**) Quantitative RT-PCR analysis of Gli1 and Gli2 mRNA expression in C2C12 cells treated with 100 μg/ml LPS (n = 3). (**d**) Representative immunoblots of Shh, Gli1, and Gli2 expression in C2C12 cells exposed to gradient LPS concentrations (0, 1, 20, and 100 μg/ml; n = 3) for 24 h, with quantification presented. (**e**) Representative images of Gli1 IF staining in C2C12 cells treated with or without LPS and its co-location analysis conducted by ImageJ (scale bar: 20 μm&50 μm). (**f**) Gli1 motif and schematic diagram of the putative binding site of Gli1 in mouse Dio3 promoter regions predicted by the JASPAR database. (**g**) Agarose gel electrophoresis map of DNA samples amplified by PCR, which are immunoprecipitated and purified by Gli1 antibody binding to C2C12 cell lysates with or without treatment of LPS or rShh and its quantification. (**h**) Quantitative RT-PCR analysis of Dio3 expression in the promoter binding site of ChIP samples in the same setting as in (g). (**i**) Immunoblotting demonstrating Dio3 expression in H293T, HepG2, and PC9 cells treated with rShh and its quantification (n = 3). (**j**) Immunoblotting demonstrating the expression of Dio3 in C2C12 cells treated with 100 μg/ml LPS or 10 μM Gant61 (n = 3) and its quantification. ^*^P < 0.05. Two-tailed Student’s unpaired t-test for (c, h, and i) or ANOVA followed by Tukey’s multiple comparisons tests for (a, b, d, g, and j). Quantitative data are represented as mean ± SD. *NS* not significant, *Shh* sonic hedgehog, *Dio3* type 3 deiodinase, *LPS* lipopolysaccharide, *GLI* GLI family zinc finger

The Shh pathway exerts its regulatory effects predominantly through downstream transcription factors, notably Gli1 and Gli2 [20]. Thus, we treated differentiated C2C12 cells with a specific inhibitor of Gli1/2, Gant61, which led to the marked attenuation of rShh-induced Dio3 expression (Figure 5a). In addition, extended Gant61 treatment hindered Dio3 activation (Figure 5b). Although Gli2 has been implicated in the regulation of Dio3 in BCC, its expression was suppressed early under LPS stimulation, whereas Gli1 expression was remarkably elevated (Figure 5c). Concentration-dependent stimulation with LPS corroborated the evident increase of Gli1 expression level rather than Gli2 under inflammatory conditions (Figure 5d). Furthermore, the pronounced nuclear translocation of Gli1 was observed after LPS exposure (Figure 5e). These findings indicate that Gli1 may predominate the early regulatory role of Dio3. In validating this hypothesis, we obtained the Dio3 promoter sequence and predicted the possible binding site of Gli1 using the JASPAR database. A specific Gli1 binding site within the core promoter region of Dio3 was found accordingly (Figure 5f). ChIP quantitative PCR agarose gel electrophoresis (Figure 5g) and ChIP RT-qPCR results (Figure 5h) corroborated the binding of Gli1 to the Dio3 promoter, with this binding being mediated by LPS and rShh. Given that the C2C12 cell line is derived from murine, the regulatory action of rShh on Dio3 was revalidated to exclude species heterogeneity across three human cell lines: H293T, PC9, and HepG2 (Figure 5i). In validating the regulatory role of Gli1 on Dio3 under inflammation conditions, Gant61 was administered to C2C12 cells with or without LPS exposure. The findings indicated that Gant61 exposure remarkably diminished Dio3 expression regardless of the LPS challenge (Figure 5j). Prolonged Gant61 exposure further attenuated the LPS-inducible effects on Dio3 (Figure S3). Collectively, these results demonstrate the direct regulatory role of Shh signaling in Dio3 expression.

### Shh signaling blockade suppressed Dio3 induction, ameliorated low T3 state, and glucose intolerance *in vivo*

**Figure 6:**
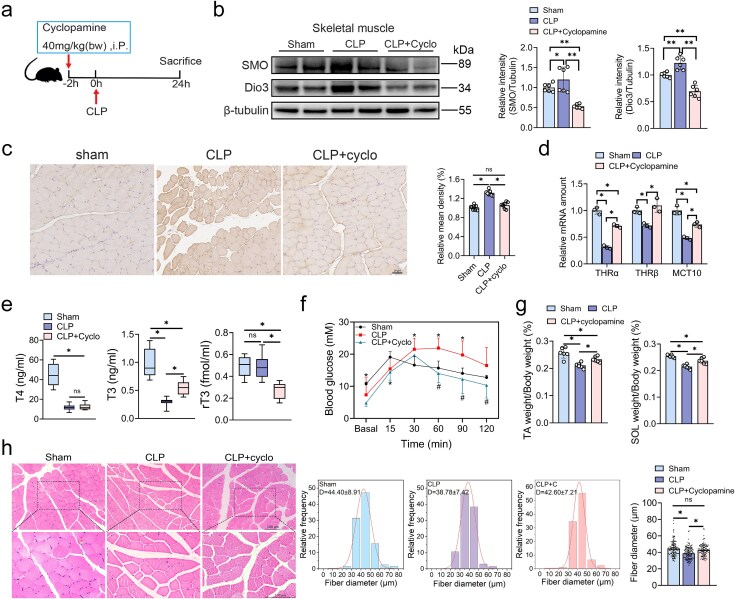
Shh signaling blockade suppressed Dio3 induction and ameliorated low T3 state and glucose intolerance *in vivo*. (**a**) A brief time-course schematic diagram of cyclopamine administration and CLP modelling. (**b**) Representative immunoblots of Dio3 and SMO in skeletal muscles in the same settings as in (a; n = 6), with quantification presented. (**c**) Representative images of Dio3 IHC staining in the same setting as in (a) and its quantification (n = 6, scale bar: 50 μm). (**d**) Quantitative RT-PCR analysis of T3-responsive genes in samples in the same setting as in (a). (**e**) Serum T4, T3, and rT3 levels in rats subjected to sham or CLP modelling or CLP and cyclopamine administration (n = 6–8) detected by RIA. (**f**) Intraperitoneal glucose tolerance test of rats treated with sham CLP, or CLP + Cyclopamine (n = 6). (**g**) Proportion of TA or soleus muscles to body weight (n = 6). (**h**) Representative images of the HE staining of skeletal muscles in the same setting as in (a, n = 6) and quantitative analysis of fiber diameters by ImageJ. ^*^P < 0.05. One-way ANOVA followed by Tukey’s multiple comparisons tests for (b, h) or one-way ANOVA followed by Tukey’s multiple comparisons tests for (c, d, e, f, and g). Quantitative data are represented as mean ± SD. *NS* not significant, *Dio3* type 3 deiodinase, *CLP* cecal ligation and puncture, *SMO* protein smoothened, *THR* thyroid hormone receptor, *T3* triiodothyronine, *T4* thyroxine, *rT3* reverse, T3, *Cyclo* cyclopamine, *TA* tibialis anterior, *SOL* soleus muscle

To further corroborate the regulatory role of the Shh pathway in Dio3 *in vivo*, we applied the specific SMO inhibitor cyclopamine to hinder Shh signaling. A schematic diagram of cyclopamine administration and CLP modelling is shown in Figure 6a. Corresponding changes in the expression level of SMO and Dio3 in skeletal muscles and lung tissues substantiated the effective repression of Dio3 expression via blockade of the Shh pathway (Figures 6b and S4). The IHC staining results also indicated repressed Dio3 expression in skeletal muscle samples from rats in the CLP + cyclopamine group (Figure 6c). Moreover, the expression of T3-responsive genes was restored by cyclopamine, indicating improved local TH responsiveness (Figure 6d). Considering that the induction of Dio3 was deeply involved in the pathogenesis of NTIS, the alterations in the TH axis were further evaluated. Based on the results, rats that received CLP modeling and cyclopamine displayed a significant reduction in rT3 levels with a concomitant elevation in T3 levels, while T4 levels remained unaltered (Figure 6e). The improved TH responsiveness in skeletal muscles may lead to a preserved glycolytic phenotype and GLUT4 function. Accordingly, the intraperitoneal glucose tolerance test results indicate that stress-induced glucose metabolic disorder was improved in the CLP + cyclopamine group (Figure 6f). In addition, the ratio of TA and SOL weight to body weight and the fiber diameter were significantly increased because of Dio3 suppression compared with the CLP group (Figure 6g and h). Collectively, pharmacological blockade of the Shh pathway effectively inhibited the induction of Dio3 in tissues, ameliorated low T3 state, improved systemic glucose metabolic disorders, and preserved muscle mass.

### IL-6 and ROS mediate Shh activation via STAT3

**Figure 7:**
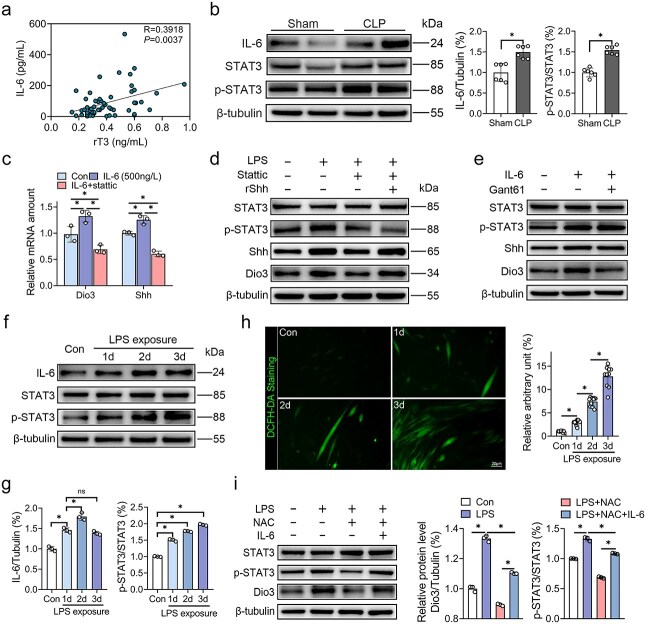
IL-6 and ROS mediate Shh activation via STAT3. (**a**) Correlation analysis between rT3 and IL-6 (n = 62). (**b**) Representative immunoblots of IL-6, STAT3, and p-STAT3 expression in skeletal muscle samples of rats subjected to sham or CLP modelling (n = 6) and its quantification. (**c**) Quantitative RT-PCR analysis of Dio3 and Shh expression in C2C12 cells treated with 500 ng/ml IL-6 or 5 μM Stattic (n = 3). (**d**) Representative western blots of Shh, Dio3, STAT3, and p-STAT3 expression in C2C12 cells treated with 500 ng/ml IL-6 or 5 μM Stattic (n = 3). (**e**) Representative western blots of Shh, Dio3, STAT3, and p-STAT3 expression in C2C12 cells treated with 100 μg/ml LPS or 5 μM Stattic or 500 ng/ml rShh (n = 3). (**f**) Representative immunoblots of IL-6, STAT3, and p-STAT3 expression in C2C12 cells treated with 100 μg/ml LPS for different time courses (n = 3). (**g**) Quantitative analysis of (**f**). (**h**) Representative DCFH-DA staining fluorescent images of C2C12 cells treated with 100 μg/ml LPS for different time courses (n = 10) and its quantification. (**i**) Representative western blots of STAT3, p-STAT3, Shh, and Dio3 expression in C2C12 cells treated with 100 μg/ml LPS or 10 mM NAC (n = 3). ^*^P < 0.05. Spearman correlation analysis for (a), two-tailed Student’s unpaired t-test and Mann–Whitney test for (b, h), or one-way ANOVA followed by Tukey’s multiple comparisons test for (c, g, and i). Quantitative data are represented as mean ± SD. *NS* not significant, *IL-6* interleukin 6, *rT3* reverse triiodothyronine, *STAT3* signal transducer and activator of transcription 3, *Dio3* type 3 deiodinase, *Shh* sonic hedgehog, *LPS* lipopolysaccharide, *NAC* N-acetylcysteine

Although the mechanisms involved in Shh signaling activation are vastly investigated in tumor research, the mechanisms driving its upregulation under septic conditions remain unclear. IL-6-STAT3 signaling not only controls Shh activation in pancreatic tumors [21], but also exerts a strong correlation with Dio3 expression [22]. Thus, we hypothesize that IL-6-STAT3 signaling stimulates the activation of the Shh pathway. Accordingly, we found a positive correlation between the levels of IL-6 and rT3 (Figure 7a). The expression level of IL-6, STAT3, and p-STAT3 in the skeletal muscle of CLP rats was also robustly increased 24 h post-CLP (Figure 7b). To confirm whether the IL-6/STAT3 pathway is involved in the regulation of Dio3 expression in skeletal muscles, we treated differentiated C2C12 cells with recombinant 500 ng/L IL-6 and 5 μM Stattic, a specific inhibitor of STAT3. IL-6 administration markedly augmented the expression levels of Dio3 and Shh; however, this upregulation was mitigated upon Stattic application, underscoring the pivotal role of STAT3 in the IL-6-driven modulation of Dio3 (Figure 7c). To elucidate the mechanism of IL-6/STAT3 in Dio3 regulation, C2C12 cells were treated with LPS, LPS + Stattic, or LPS + Stattic + rShh. The findings indicate that Stattic suppressed the upregulation of Shh and Dio3, while additional administration of rShh restored Dio3 expression without affecting p-STAT3 levels (Figure 7d), thereby confirming that the Shh pathway operates downstream of STAT3. In addition, the inducive effect of IL-6 on Dio3 was abolished by Gant61, indicating that the regulatory role of IL-6/STAT3 on Dio3 was dependent on Shh signaling (Figure 7e).

Although IL-6 is an initial activator of STAT3, it is likely insufficient to sustain the activation of STAT3 [21]. Concordant with this perspective, our observations revealed that a 3-day LPS treatment of C2C12 cells remarkably increased IL-6 expression on the initial day, followed by a modest rise on the second day and a subsequent reduction on the third day. Concurrently, the ratio of p-STAT3 to STAT3 exhibited a consistent upwards trajectory throughout the exposure (Figure 7f and g). Given that ROS is deeply involved in the pathology of NTIS [23] and serves as a potent driver of STAT3 [24], we hypothesize that ROS may induce the expression of Dio3 by sustaining STAT3 activation. Thus, we measured ROS levels and discerned a significant elevation consistent with the duration of LPS exposure (Figure 7h). In addition, scavenging ROS by using 5 mM N-acetylcysteine (NAC) markedly reduced STAT3 phosphorylation and attenuated the expression level of Shh and Dio3. Interestingly, this inhibitory effect was attenuated by 1000 ng/L IL-6 administration (Figure 7i). Thus, ROS may partially induce Dio3 expression by activating STAT3.

Collectively, these results indicate that IL-6/ROS mediates the induction of Shh signaling by sustaining the activation of STAT3, thereby accelerating the expression of Dio3 through Gli1’s binding to its promoter.

## Discussion

In this study, we demonstrated that baseline rT3 is a better biomarker for predicting mortality and reflecting organ function than T3 and fT3. In *in vivo* study, we observed a notable upregulation of Dio3 expression in the lung and skeletal muscles during the incipient stages of sepsis. The targeted inhibition of Dio3 restores TH responsiveness and ameliorates sepsis-induced metabolic disruption in skeletal muscles, which leads to preserved muscle mass. The activation of Dio3 is facilitated by the STAT3/Shh/Gli1 axis. The selective blockade of the Shh pathway not only restored T3 responsiveness in muscles, but also improved the systemic TH axis and glucose intolerance. Mechanistically, IL-6 and ROS synergistically sustained the phosphorylation of STAT3 during early sepsis, thereby initiating the activation of the Shh pathway, by which the binding of Gli1 is potentiated to the Dio3 promoter. Thus, we proposed a new therapeutic strategy in which targeting a STAT3/Shh/Gli1-regulated pathway of Dio3 ameliorates metabolic disarrangements in skeletal muscles under septic conditions.

rT3 has long been regarded as an inactive form of the TH axis with little biological function; however, it can robustly inhibit Dio2 activity, thereby impeding local TH action [25]. Increased rT3 levels have been observed in various physiological and pathological conditions, including myocardial infarction, hepatitis, and hepatic cirrhosis [26]. Notably, increased T3/rT3 ratio is deeply associated with insulin resistance [27]. The theory of rT3 being a marker for acute metabolic dysregulation is recognized. In the present study, rT3 levels were elevated in 54.84% of our patient population, which is consistent with the findings of previous research [28]. The observed augmentation in rT3 levels can be attributed principally to two underlying mechanisms: first, the attenuation of clearance resulting from repressed Dio1 activity; second, the increased synthesis stemming from the upregulation of Dio3 [18]. Thus, increased illness severity may result in an expedited inactivation of the TH axis, thereby facilitating an incessant conversion of T4 into rT3. Moreover, we found that rT3 levels are closely associated with organ function, which can be attributed to the direct link between TH actions and the tissue metabolic rate as well as mitochondrial function.

The expression of Dio3 varies across different tissues. Based on previous reports, liver Dio3 mRNA expression is robustly reduced in chronic and acute inflammation, while a study using a toxin-induced hepato-necrosis mouse model with liver Dio3 deficiency achieved early restoration of peripheral T3 levels [29, 30]. Although Dio3 mRNA expression in skeletal muscle remains unaltered when facing acute inflammation, its expression increased robustly in chronic inflammation [31]. However, data from biopsy samples of critically ill patients indicated that the Dio3 activity of the liver and muscles is strongly increased [12]. Here, we evaluated the expression of Dio3 at the protein level in six major organs. We found a notably reduced expression of Dio3 in the liver, kidney, and intestinal tissues 24 h after CLP modelling, aligning with the findings of prior research and possibly elucidating why hepatic knockout of Dio3 failed to restore the T3 levels within 24 h [30, 32]. The alteration of Dio3 expression in the kidney is similar to Reiser’s study, which demonstrated reduced Dio3 expression upon injury in podocytes [33]. In contrast, an upregulation of Dio3 was observed in the heart, lung, and skeletal muscles. Although Dio3-induced cardiac TH axis inactivation might affect cardiac function and lead to myocardial remodeling, it may also play a protective role during the acute phase of illness [34]. Although little research focused on the expression of Dio3 in the lung upon inflammation, the increased expression level and activity of Dio3 in skeletal muscle were repeatedly affirmed. In addition, our results demonstrated a more pronounced alteration of Dio3 in skeletal muscle cells in comparison with lung PC9 cells. Similar alterations in the skeletal muscle TH axis were evident in starvation-induced NTIS, where the effects of starvation on the lungs were minimal compared with those on skeletal muscle. Given that skeletal muscle constitutes a larger proportion of body mass and plays a critical role in metabolic processes, we propose that the early elevated rT3 may primarily emanate from skeletal muscles.

Skeletal muscle is an organ that is most vulnerable to acute stress. Upon acute stress, its functions shift from energy storage, locomotion, and metabolic regulation to energy source providers serving as an amino acid pool for hepatic gluconeogenesis [35]. The low T3 state induced by fasting illustrates that even mild stress can disrupt the metabolic equilibrium in skeletal muscles. Given the vital role of TH in maintaining myofiber type [36], we demonstrate that Dio3 inhibition prevents sepsis-induced fast-to-slow myofiber conversion, which preserves skeletal muscles in a glycolytic phenotype. Moreover, given that TH plays a key role in maintaining the function of Glut4 [37], restored TH action in skeletal muscles may remarkably improve systemic glucose metabolism. Hyperglycemia is frequently observed in critically ill patients with higher blood glucose when facing nutritional therapy [38]. Thus, targeted inhibition of Dio3 in skeletal muscles can be an effective method to treat stress-induced hyperglycemia. Furthermore, the inhibition of Dio3 induced genes involved in mitochondrial biogenesis and lipid metabolism, which may help to counteract the ICU-acquired weakness by preserving mitochondrial function [39]. In addition, TH is strongly involved in the activation of AKT signaling in skeletal muscles [40]. Hence, the balance between protein synthesis and proteolysis can be rectified by inhibiting Dio3, which may protect muscle mass against sepsis-induced muscle atrophy.

The mechanisms of Dio3 induction have been vastly investigated in tumors and ischemic injury, involving pathways such as Shh, MAPK, and Hif1α. Among these signaling pathways, a significant overlap in functionalities between the Shh pathway and Dio3 was observed [41]. Research into BCC revealed that Shh signaling governs the modulation of Dio3 through Gli2 [19]. Additional research has also demonstrated that rShh can induce Dio3 expression in cultured skeletal satellite cells [13]. Thus, the Shh pathway serves as an upstream regulator influencing Dio3 expression. Although Gli2 has been identified as a principal regulator of Dio3 in oncogenic contexts, evidence suggests that Gli1 exhibits a more pronounced responsiveness to inflammatory stimuli. Zhang Ping and colleagues revealed an inflammation-induced increase of Gli1 expression in hematopoietic stem cells [42], while Bibo Ke demonstrated a marked upregulation of Gli1 in mesenchymal stem cells in a mouse model of bacterial liver injury [43]. We further demonstrated that LPS exposure induced a significant elevation in Gli1 expression, which governs the induction of Dio3 transcriptionally.

Previous research has also established the profound involvement of IL-6 in Dio3 regulation. STAT3, a principal transcription factor downstream of the IL-6 signaling pathway, plays a vital role in the early stages of sepsis [44]. Studies focusing on tumors have discerned that STAT3 is essential for the activation of Shh signaling [45]. Notably, ROS plays an important role in sustaining STAT3 activation. Wörmann observed more potent maintenance of STAT3 activation by ROS in pancreatic cancer tissue [21]. Lan Wu also demonstrated that ROS, through the activation of the JAK2/STAT3 pathway, exhibits cardioprotective function in early cardiac ischemia–reperfusion injury [46]. Our results indicate that ROS clearance through NAC lowers the phosphorylation of STAT3 and attenuates the expression of Shh and Dio3.

Notably, the Shh/Gli1 pathway predominantly regulates Dio3 in skeletal muscles and lung tissues upon early sepsis. However, whether this regulatory dominance extends to other tissues remains to be investigated. Based on previous reports, the MAPK pathway can induce Dio3 expression by upregulating Gli1 through non-canonical pathways [47]. Moreover, Warner S. Simonides showed that hypoxia increases Dio3 expression in neurons, cardiomyocytes, and liver cells, under the regulation of Hif1a [14]. Considering the significant upregulation of Hif1a across various tissues in sepsis, the role of Hif1a in NTIS cannot be discounted. However, our research group focuses on metabolism-related issues in sepsis, particularly on the critical role of skeletal muscles in sepsis-induced metabolic dysregulation. Therefore, we primarily investigated Dio3 regulation in skeletal muscles. Recent studies have suggested that the global inhibition of Dio3 might have adverse effects, as Dio3 is essential for maintaining immune cell function, and its inhibition can impair neutrophil bacterial clearance [48]. Consequently, we aim to utilize skeletal muscle–targeted drug systems in future studies, as tissue-specific drug delivery could mitigate the side effects associated with systemic Dio3 inhibition.

This study has also some limitations. First, this study recruited a relatively limited number of patients. Second, the skeletal muscle–targeted Dio3 knockout murine model should be more convincing in confirming the vital role of Dio3 in preserving metabolic balance not only in TA but also in all skeletal muscles. Third, deiodinase activity is a potent parameter in reflecting enzyme function, although immunoblotting was ensured as an effective method of evaluating Dio3 expression.

## Conclusions

In this study, an in-depth elucidation of mechanisms involved in the activation of Dio3 was conducted, and the pivotal role of Dio3 in facilitating metabolic alterations in sepsis was delineated. The targeted inhibition of Dio3 ameliorated sepsis-induced metabolic dysregulation in skeletal muscles. Our findings provided novel solutions for clinical interventions aimed at rectifying metabolic disarrangements in sepsis.

## Abbreviations

NTIS, The Non-thyroidal illness syndrome; Dio3, type 3 deiodinase; Shh, Sonic Hedgehog; ROS, reactive oxygen species; STAT3, signal transducer and activator of transcription 3; TH, thyroid hormones; IL-6, Interleukin 6; PCT, Procalcitonin; TSH, thyroid stimulating hormone; T3, triiodothyronine; rT3, reverse T3; T4, thyroxine; Dio2, type 2 deiodinase; TRH, thyrotropin-releasing hormone; APACHE II, Acute Physiological and Chronic Health Evaluation II; SOFA, Sequential Organ Failure Assessment; ROC, receiver operating characteristic; LPS, lipopolysaccharide; SMO, smoothened; Gli1, Glioma Associated Oncogene Family Zinc Finger 1; BCC, basal cell carcinomas; IF, immunofluorescence; IHC, Immunohistochemistry; ChIP, chromatin immunoprecipitation; NAC, N-acetylcysteine; THR, thyroid hormone receptor; MCT10, Monocarboxylic Acid Transporter 10.

## Author contributions

Gang Wang (Conceptualization [lead], Data curation [lead], Formal analysis [lead], Investigation [lead], Methodology [lead], Resources [equal], Software [equal], Validation [lead], Visualization [lead]), Tao Gao (Conceptualization [lead], Data curation [lead], Formal analysis [lead], Investigation [lead], Methodology [lead], Project administration [equal], Resources [lead], Validation [lead]), Yijiang Liu (Data curation [equal], Formal analysis [equal], Investigation [equal], Validation [equal]), Jianfeng Duan (Funding acquisition [equal], Supervision [equal], Validation [equal]), Huimin Lu (Data curation [equal], Validation [equal]), Anqi Jiang (Data curation [equal], Investigation [equal]), Yun Xu (Data curation [equal], Investigation [equal]), Xiaolan Lu (Supervision [equal], Validation [equal]), Xiaoyao Li (Funding acquisition [equal], Supervision [equal], Validation [equal]), Yong Wang (Methodology [equal], Project administration [equal], Supervision [equal]), Wenkui Yu (Conceptualization [lead], Methodology [lead], Project administration [lead], Resources [lead], Supervision [lead]¸ Validation [lead], Visualization [lead]).

## Ethics approval and consent to participate

All procedures of the animal experiments were approved by the Animal Research Committee of Nanjing University Medical School (2022AE02004). All clinical data and skeletal muscle samples were collected with written informed consent from patients and following the guidelines approved by the ethics boards of the Nanjing Drum Tower Hospital (Ethics number: 2023–427-03).

## Consent for publication

All authors have approved the publication of this manuscript.

## Supplementary Material

Supplementary_figs_and_tables_tkae066

## Data Availability

The raw data of RNA-seq were deposited in SRA with the accession number: PRJNA1071726. Other data of the study are available from the corresponding author upon reasonable request.
