# Supplementary material for: Type 3 deiodinase activation mediated by the Shh/Gli1 axis promotes sepsis-induced metabolic dysregulation in skeletal muscles
Source: Burns Trauma. 2025 Jan 28;13:tkae066. doi: 10.1093/burnst/tkae066 (PMC11773416; doi:10.1093/burnst/tkae066)
Supplement: Supplementary_figs_and_tables_tkae066 [file supplementary_figs_and_tables_tkae066.docx]

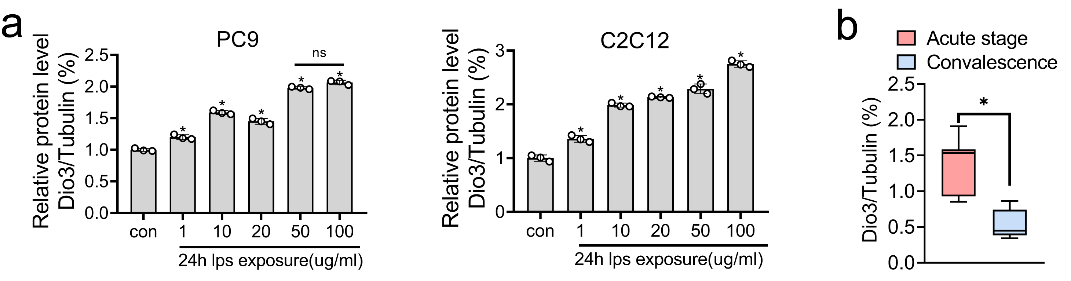


**Supplementary Figure S1.** Dio3 expression increased mainly in skeletal muscle and lung tissues upon early sepsis. (**a**) Quantitative analysis of Immunoblotting demonstrating expression of Dio3 in PC9 and C2C112 cells exposed to gradient LPS concentrations for 24h (0, 1, 10, 20, 50, 100 µg/mL) (n=3). (**b**) Quantitative analysis of immunoblotting demonstrating expression of Dio3 in human skeletal muscle biopsy samples. *, *P* < 0.05; NS, not significant. One-way ANOVA followed by Tukey’s multiple comparisons test was used for (**a**) or Mann Whitney test for (**b**). Quantitative data are represented as mean ± SD. Dio3, type 3 deiodinase; LPS, Lipopolysaccharide.


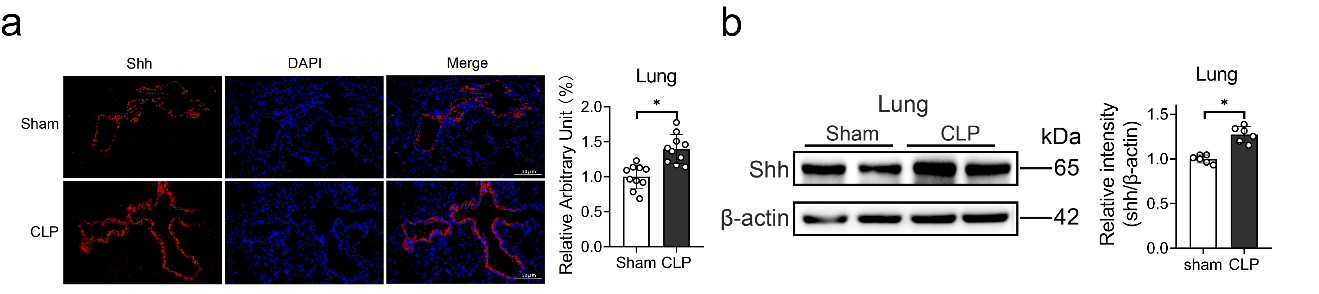
**Supplementary Figure S2.** Shh signaling is involved in the reactivation of Dio3**.** (**a**) Representative images of immunofluorescence (IF) staining of Shh in the pulmonary tissues (scale bar, 50 µm) and quantification of Shh-positive area per selective field in rats with or without CLP modeling, respectively (n=6). (**b**) Representative immunoblots of Shh expression in pulmonary tissues of control rats or rats subjected to CLP modeling and its quantification (n=6). *, *P* < 0.05. Two-tailed Student’s unpaired t-test was used for statistical analysis. Quantitative data are represented as mean ± SD. Shh, Sonic Hedgehog Protein; CLP, cecal ligation and puncture.


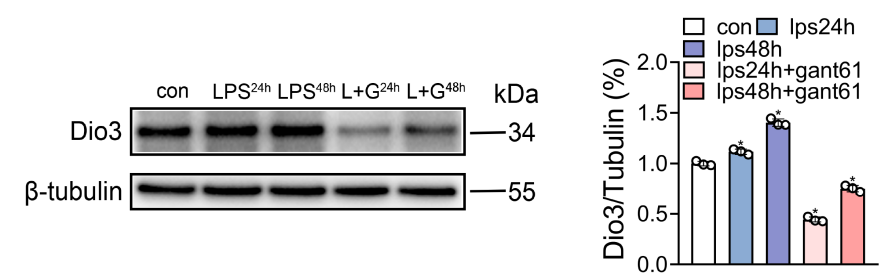


**Supplementary Figure S3.** Shh pathway regulates Dio3 expression via Gli1 in myoblasts under an inflammatory backdrop. Immunoblotting demonstrating expression of Dio3 in C2C12 cells treated with 100 µg/mL LPS or 10 µm Gant61 for 24h or 48h (n=3) and its quantification. *, *P* < 0.05. One-way ANOVA followed by Tukey’s multiple comparisons test was used for statistical analysis. Quantitative data are represented as mean ± SD. Dio3, type 3 deiodinase; LPS, Lipopolysaccharide; G, Gant61.


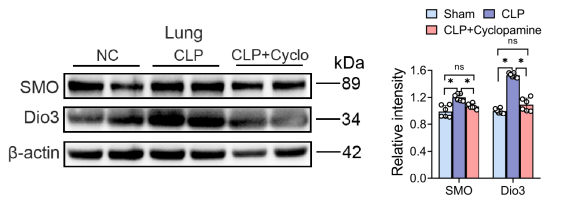


**Supplementary Figure S4.** Shh signaling blockade suppressed Dio3 induction, ameliorated low T3 state and glucose intolerance in vivo. Quantitative analysis of representative western blots of SMO and Dio3 expression in lung samples from rats subjected to sham or CLP modeling or both CLP and cyclopamine administration (n=6). *, *P* < 0.05; NS, not significant. Kruskal-Wallis test and One-way ANOVA followed by Tukey’s multiple comparisons test was used for statistical analysis. Quantitative data are represented as mean ± SD. Dio3, type 3 deiodinase; SMO, Protein Smoothened; Cyclo, cyclopamine; CLP, cecal ligation and puncture.

Table S1. Primary antibodies list

| Reagent or resource | Source | Identifier |
| --- | --- | --- |
| Antibodies | | |
| Anti-Dio3 | Abclonal | Cat# A6900; RRID: AB_2767459 |
| Anti-Shh | Abclonal | Cat#A12695; RRID: AB_2861675 |
| Anti-Gli1 | Santa Cruz | Cat#sc-515751; RRID: AB_2934097 |
| Anti-Gli2 | Abclonal | Cat#A6510; RRID: AB_2767106 |
| Anti-SMO | proteintech | Cat#66851-1-Ig; RRID: AB_2882191 |
| Anti-IL-6 | Beyotime | Cat#AF0201; RRID: AB_2833285 |
| Anti-STAT3 | proteintech | Cat# 10253-2-AP; RRID: AB_2302876 |
| Anti-*p-*STAT3 | CST | Cat# 9131S; RRID: AB_331586 |
| Anti-FoxO1 | CST | Cat#2880S; RRID: AB_2106495 |
| Anti-*p-*FoxO1 | CST | Cat#84192S; RRID: AB_2800035 |
| Anti-mTOR | CST | Cat#2983S; RRID: AB_2105622 |
| Anti- *p-*mTOR | CST | Cat#5536S; RRID: AB_10691552 |
| Anti-Pan-AKT | CST | Cat#4691T; RRID: AB_915783 |
| Anti-*p-*AKT | Santa Cruz | Cat#sc-514032; RRID: AB_2861344 |
| Anti-Trim63 | proteintech | Cat#55456-1-AP; RRID: AB_11232209 |
| Anti-FBXO32 | proteintech | Cat#67172-1-Ig; RRID: AB_2882468 |
| Anti-Glut4 | Abclonal | Cat#A7637; RRID: AB_2768149 |
| Anti-β-tubulin | proteintech | Cat# 66240-1-Ig; RRID: AB_2881629 |
| Anti-β-actin | proteintech | Cat#66009-1-Ig; RRID: AB_2687938 |
| HRP, Goat Anti-Rabbit IgG | Abbkine | Cat# A21020; RRID: AB_2876889 |
| HRP, Goat Anti-Mouse IgG | Abbkine | Cat# A21010; RRID: AB_2728771 |

Table S2. Primers used for qRT-PCR

| Mouse gene | Primers (5’ → 3’) |
| --- | --- |
| Dio3 | FWD: GTTTTTGGCTTGCTCTCAGG  REV: CAACAAGTCCGAGCTGTGAA |
| Shh | FWD: AAAGCTGACCCCTTTAGCCTA  REV: TTCGGAGTTTCTTGTGATCTTCC |
| Gli1 | FWD: CCAAGCCAACTTTATGTCAGGG  REV: AGCCCGCTTCTTTGTTAATTTGA |
| Gli2 | FWD: CAACGCCTACTCTCCCAGAC  REV: GAGCCTTGATGTACTGTACCAC |
| β-actin | FWD: GTGACGTTGACATCCGTAAAGA  REV: GCCGGACTCATCGTACTCC |
| Rat gene | Primers (5’ → 3’) |
| THRα | FWD: GCTTCTTTCGCCGTACAATC  REV: ACTGATTCCGGGTGATCTTG |
| THRβ | FWD: TTTCCTGTTGGCCTTTGAAC  REV: AGGTCCGTCACCTTCATCAG |
| MCT10 | FWD: GACCGCCTATGCCGTGTGGG  REV: CGGCCGAAGAGAAGCCGTCC |
| PGC1a | FWD: GTCAACAGCAAAAGCCACAA  REV: GTGTGAGGAGGGTCATCGTT |
| NRF-1 | FWD: CAACAGGGAAGAAACGGAAA  REV: GTGGCTCTGAGTTTCCGAAG |
| TFAM | FWD: CTGATGGCCATTACATGTGG  REV: AAAGCCCGGAAGGTTCTTAG |
| Myh4 | FWD: TGCCAAGTCCATCCCGAAGT  REV: GGTCACCCGCATCAACCAGC |
| Myh7 | FWD: TGGATCAGGACAAGAAGGTGC  REV: AGTCCTTCTTTTTGAGTCGCT |
| CPT1b | FWD: ATCGAACGTGCTGCTTTCTT  REV: ATTTGCCGTAGAGGCTGAGA |
| CD36 | FWD: TTTCCTCTGACATTTGCAGGTCTA  REV: AAAGGCGTTGGCTGGAAGAA |
| SCAD | FWD: TGCCCTATGTTTCGCACCTC  REV: TTCAATGCCCATCATCCCTT |
| β-actin | FWD: ACCTTCTACAATGAGCTGCG  REV: CCTGGATAGCAACGTACATGG |
| Human gene | Primers (5’ → 3’) |
| Dio3 | FWD: ATCCTCGACTACGCGCAAG  REV: GGGATGATGTAGGGAGAGTCC |
| Shh | FWD: CTCGCTGCTGGTATGCTCG  REV: ATCGCTCGGAGTTTCTGGAGA |
| Gli1 | FWD: GGGTGCCGGAAGTCATACTC  REV: GCTAGGATCTGTATAGCGTTTGG |
| Gli2 | FWD: CATGGAGCACTACCTCCGTTC  REV: CGAGGGTCATCTGGTGGTAAT |
| GUSB | FWD: CACCAGGGACCATCCAATACC  REV: GCAGTCCAGCGTAGTTGAAAAA |
